# Joint coordination constraints using an upper limb exoskeleton impact novel skill acquisition

**DOI:** 10.1017/wtc.2025.10028

**Published:** 2025-10-27

**Authors:** Keya Ghonasgi, Reuth Mirsky, Adrian M. Haith, Peter Stone, Ashish D. Deshpande

**Affiliations:** 1 Department of Mechanical Engineering, The University of Texas at Austin, Austin, TX, USA; 2 Department of Computer Science, Tufts University, Boston, MA, USA; 3 Department of Neurology, Johns Hopkins University, Austin, TX, USA; 4 Department of Computer Science, The University of Texas at Austin, Austin, TX, USA; 5 Sony AI, Austin, TX, USA

**Keywords:** exoskeletons, human motor control, physical human robot interactive controllers, human–robot interaction

## Abstract

Robotic exoskeletons offer the potential to train novel motor skill acquisition and thus aid physical rehabilitation. Our prior work demonstrated that individuals converge to certain kinematic coordinations as they learn a novel task. An upper-limb exoskeleton controller that constrains individuals to this known coordination was also shown to significantly improve straight-line reaching task performance. This paper studies the impact of variations of this controller on novel skill acquisition. We quantify learning under three variations of the intervention (each group with *N* = 10 participants) against a control group (*N* = 13). Our results show that introducing any constraint during learning can hinder the learning process, as this alters the task dynamics that lead to success. However, when presented with a personalized constraint, participants still learn. When presented with a task-specific constraint, rather than a personalized one, participants cannot overcome the differences in the training and target task, suggesting exoskeleton-based training interventions should be personalized. The changes in kinematic behaviors during learning further suggest that participants do not have a statistically consistent performance. While participants respond more to exoskeleton intervention, others may not respond in short training sessions, necessitating further analysis of how strong a response can be encouraged. Our findings emphasize the need for further study of the effects of exoskeleton intervention for motor training and the potential need for personalization.

## 1. Introduction

Wearable robotic systems are becoming more capable of safe and effective interactions with their wearers. These devices have demonstrated the ability to assist in functional task performance and even bring about the adaptation of known movement behaviors (Young & Ferris, 2016; Proietti et al., 2022). The same devices offer the potential to not only assist the wearer (Yun et al., 2020; Franks et al., 2021; Ding et al., 2023; Nasr et al., 2023) but also train them to improve performance and learning (Rose et al., 2021). Specifically, robotic devices such as exoskeletons could train novel motor behaviors through physical interaction (Heuer & Luettgen, 2015) with the eventual goal of enabling rehabilitation post neurological injury (de Oliveira et al., 2019; He et al., 2021; Hailey et al., 2022). Such robotic motor training could have several applications, including sports training, surgical skills training, physical rehabilitation, and teaching individuals with amputation novel motor behaviors to better control prosthetic devices.

Motor training aims to improve the efficiency of motor learning, by which humans learn novel motor tasks. This learning process is difficult to quantify and has long been studied by movement scientists (Krakauer et al., 2019). Testing motor training interventions is challenging as humans have high variability in their learning abilities and behavior when presented with novel motor tasks. Prior studies have largely focused on learning tasks in static environments where the learner’s movements fully control the environment. For example, reaching a static target with visual or motor perturbations (Losey et al., 2016; Elangovan et al., 2017) has been studied extensively to understand how human behaviors adapt to such disturbances. However, learning in such tasks only requires movements that participants (at least those without impairments) can already generate and, as such, do not induce learning of genuinely new movement patterns. Instead, these studies observe motor adaptation to the task or exoskeleton environment, also referred to as transformation learning (Heuer & Luettgen, 2015). Some prior work has evaluated learning of novel movement trajectories within a static environment (Sans-Muntadas et al., 2014; Wu et al., 2014). However, research on exoskeleton interaction modes that elicit improvements in human performance through motor learning rather than task assistance remains sparse. Further, few studies have considered learning dynamic tasks, where participants must not only learn a motor behavior but also react to the environment’s dynamics (Bazzi & Sternad, 2020).

This paper explores the motor training potential of an exoskeleton interaction mode that has been shown to assist in known task movements. Two key features of the current study differentiate it from prior work. First, we focus on *de Novo* learning (Krakauer, 2006) of dynamic motor tasks rather than the more commonly studied reaching to static targets or trajectory following. Second, we explore intervention modes that interact with the participant in a time-independent manner, thus allowing the wearer to control their movement and learning. Such control schemes enable the exploration of the available motor landscape, necessary in the early stages of learning (Kleim & Jones, 2008; Schmidt et al., 2018) while encouraging the learner to remain close to a desired behavior. Our time-independent interaction framework, joint angle coordination control (Ghonasgi et al., 2023b), has previously been shown to assist in reaching static targets but has not been explored as a mode for novel motor skill acquisition. Thus, *we aim to identify the opportunities and challenges presented by such an assistive interaction mode in motor skill training for novel dynamic tasks.* Specifically, we explored three hypotheses: *H1) Participants will be able to perform and learn the dynamic task under different coordination-based constraints during training*, *H2) Coordination interventions may be designed to impede or assist the learning process*, and *H3) Individuals may need personalized training interventions to support learning.* Our findings, in line with those from prior literature, suggest that these interventions can indeed influence motor learning outcomes, and underscore the potential benefits of personalized approaches.

The following section (section 2) presents prior work related to motor learning and robotic interventions for motor training. Next, we present the intervention control, experimental protocol, and analysis used in this paper (section 3). We then present our results (section 4) demonstrating the feasibility of using coordination-based intervention to modulate task difficulty and the relative effects of training with such interventions on learned behavior. These results are discussed (section 5) and the relative advantages and disadvantages of the proposed intervention design. We also discuss the potential need for individualized training environments based on our observations. The paper concludes (section 6) with limitations and potential future directions for this work.

## 2. Related works and motivation

In their reviews of motor learning, Krakauer (2006) and Krakauer et al. (2019) consider the challenges of studying the acquisition of novel motor skills. The authors make a case for interactive learning where the novelty of the task, high salience, and participant engagement are key features of a good motor training intervention. We explore the learning of a salient and novel motor task based on the Japanese ball-and-cup toy, Kendama (Ghonasgi et al., 2023a). We use this task environment for further evaluation of a wearable robotic system as a platform for motor training intervention. Krakauer (2006) also suggest that teaching task behaviors through trajectory or end-effector-based assistance are likely to fail as there is insufficient control offered to the learner. However, an intervention that involves active participation of the learner, where they drive the movement and the interaction, may be more successful for motor training.

In prior work, we presented such an intervention for the assistance of a point-to-point reaching task in both the joint-space and the end-effector space (Ghonasgi et al., 2023b). The core idea of this intervention is that it targets joint coordination rather than time-dependent movement trajectories. This idea is motivated by the findings that the consistent use of joint coordination behaviors has been suggested as an indicator of motor learning (Bockemühl et al., 2010; Averta et al., 2019; Huang et al., 2021; Khanafer et al., 2021; Pei et al., 2021).

Coordination-based control has been proposed as an assistive exoskeleton interaction mode for walking (Vallery et al., 2007; Hassan et al., 2015) and functional task assistance (Ghonasgi et al., 2023b). Proietti et al. (2017) further demonstrated that, through joint-velocity coordination-based training, participants learned to modify their known joint coordination behaviors for a static reaching task with some lasting effect beyond training. However, as reaching movements are typically known behaviors, and the study did not involve learning to dynamically interact with the environment to accomplish a goal, the observed changes constitute adaptation rather than novel motor skill acquisition. Dynamic interactions may also be achieved through adaptation, but learning a dynamic unknown task environment may be an effective platform for the study of motor learning. Although coordination-based assistance may be beneficial for known task performance, it remains unclear how a similarly designed robotic intervention will affect the learning of a novel dynamic motor task.

The study presented in this paper is designed to test the effect of a coordination-based robotic intervention on novel motor skill acquisition on a dynamic task. Our initial implementation of the coordination-based intervention was designed to be assistive for a static reaching task (Ghonasgi et al., 2023a) performed while wearing a bimanual upper-limb rehabilitation robot, the Harmony Exoskeleton (Kim & Deshpande, 2017). Specifically, we use a known reference behavior to construct a desired coordination that was imposed during testing. We found that the intervention improved the accuracy of straight-line reaching movements as a consequence of maintaining the imposed joint coordination behavior. For a novel dynamic task, a novice is unlikely to succeed during their initial attempts, and thus may not be using success-correlated coordination compared to a reference behavior identified from expert data. Thus, while constructing a coordination behavior using these initial attempts to personalizes the assistance, this approach may not result in an assistive coordination control mode. In addition to this approach, we also consider a second participant-generalized approach to constructing the training coordination. Specifically, we use data from a control group of participants who learn the Kendama task without any intervention to define a task-specific, across-participant desired coordination behavior. We previously identified such behaviors to be indicative of expert performance and aim to teach this behavior to novice learners directly to increase the efficiency of learning through interaction (Ghonasgi et al., 2023a). Lastly, an additional intervention is designed to test whether participants can be constrained away from the task-specific coordination behavior.

The goal of the study presented in this paper is to evaluate the *feasibility* and the *effect* of time-independent joint-coordination-based training interventions on dynamic motor skill acquisition. Toward this evaluation, we use the Harmony Exoskeleton to study how novice participants learn to perform the dynamic Kendama task. Based on prior literature (Crocher et al., 2012; Proietti et al., 2017), we hypothesize that *participants will be able to perform the dynamic task during the coordination-based intervention introduced by the robot (feasibility).* However, at the same time, we expect that *certain interventions may introduce increased complexity to the task making it harder to learn (effect)* (Sans-Muntadas et al., 2014; Heuer & Luettgen, 2015). We also hypothesized that *participants who were trained with the same coordination-based intervention would learn the coordination behavior as a result of the training (effect).*

## 3. Methods

Below, we describe the Harmony exoskeleton and the control modes implemented on the device to study their effect on motor learning. The experimental protocol, outcome measures, and statistical analysis are explained as well.

### 3.1. The harmony exoskeleton and the Kendama task

The Harmony exoskeleton (Kim & Deshpande, 2017), a bi-manual upper limb rehabilitation robot, has seven degrees of freedom in each arm: shoulder elevation/depression, shoulder protraction/retraction, shoulder abduction/adduction, shoulder internal/external rotation, shoulder flexion/extension, elbow flexion/extension, and forearm pronation/supination. This robot has been explored as a platform for rehabilitation (de Oliveira et al., 2019; Hailey et al., 2022) and to observe motor learning as humans learn novel dynamic tasks while wearing the exoskeleton (Ghonasgi et al., 2022; Ghonasgi et al., 2023a). In the current study, the exoskeleton is torque-controlled to impose coordination-based constraints on novices as they learn a novel dynamic task based on the Japanese ball-and-cup toy Kendama. It should be noted that the Harmony exoskeleton does not allow flexion and extension of the wrist. As a consequence, all participants using the exoskeleton for the Kendama task were restricted and could not use their wrists. This restriction changes the movement behavior that participants use for the task with the exoskeleton compared to when they do not wear it. However, to maintain consistency, all participants performed the task with the wrist constraint. This allows direct comparison across different interaction groups.

**Figure 1.**
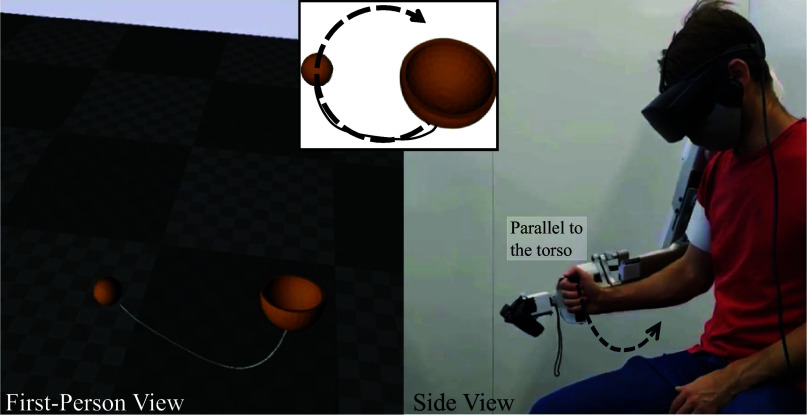
The virtual Kendama task: the left half of the image shows the virtual reality environment as observed by the participant; the right half shows a participant wearing the Harmony exoskeleton and playing the Kendama task in virtual reality. The inset shows the task strategy participants are verbally instructed to follow. The participant is instructed to move the controller parallel to their frontal plane.

The goal of the Kendama task is to swing a ball attached to a cup through a string and to catch it in the cup. A virtual simplified version of the Kendama task is used in this study (Ghonasgi et al., 2023a). The game is designed using Unreal Engine 4 and played wearing an Oculus Rift headset. The task requires the learner to manipulate the motion of the cup to control a ball attached to the cup through a string. The goal of the task is to swing the ball into the cup. To allow comparison across participants for similar learned behaviors, we explicitly instruct participants to perform the task by using arm movements parallel to their torso, in the frontal plane. Participants are verbally instructed to use this *frontal-plane* strategy to reduce the variability of task instruction across participants and to ensure the learning of comparable movement behaviors. The task and the frontal-plane strategy are depicted in Figure 1. Each movement was found to be under 4 s and was automatically cut using the velocity of the end-effector (Ghonasgi et al., 2023a). Specifically, the peak of the end-effector velocity was identified and the movement was cut such that the start was 1 s before the peak and the end was 2 s after the peak. Centering around peak-end-effector velocity and cutting attempts to a uniform length allows for comparison of the kinematic coordination for discrete attempts. The exoskeleton control runs at 200 Hz while the VR game is set to run at 60 Hz frame rate.

Learning of novel tasks has been shown to be correlated with learning of kinematic coordination behaviors (Averta et al., 2019; Ghonasgi et al., 2023a). In keeping with the high-level goal of this study, to explore the feasibility and effect of joint-coordination-based constraints on dynamic task learning, we use the Harmony exoskeleton to constrain participants’ movements as they learn to perform the dynamic Kendama task. Specifically, we extract task and participant-specific coordination behaviors and test the effect of training with these as motor constraints.

### 3.2. Kinematic coordination identification

For a given human–robot interactive movement, we can identify the kinematic coordination using principal component analysis (PCA) on the joint angle signals. Specifically, we construct a matrix 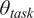 of size 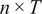 where 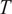 is the number of time-steps of data collected for the movement, and 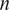 is the number of degrees of freedom of the robot for each movement for which we wish to identify the kinematic coordination. In the case of the Harmony exoskeleton, 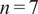 .(1)

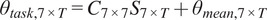



The matrix 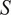 encodes the time-dependent behavior while the matric 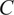 encodes the time-independent behaviors of the joints of the robot. Further, each component in 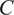 corresponds to a coordination vector along which the 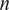 joints of the robot are coordinated during the movement. This analysis can be applied to every attempt at the virtual Kendama task.

The first vector in the identified components, the principal coordination, encodes the joint coordination responsible for the largest variability in the movement. For tasks performed with the Harmony exoskeleton, this process yields a seven-dimensional principal coordination for every attempt of the task. Note that these task attempts need not all be for one participant. Comparing the principal coordination across different attempts for an individual and across individuals allows us to construct individual-specific and task-specific coordination behaviors. Specifically, we use unsupervised k-means clustering to cluster the 7-dimensional data points across any 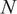 attempts (whether for a given individual or across individuals). The number of clusters is selected automatically by detecting the optimal number of clusters that describe the data well while avoiding overfitting. In this study, we cluster the data into different numbers of clusters, 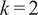 to 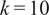 , and identify the elbow in the curve plotting change in clustering distance as the number of clusters is increased. This elbow represents the point where increasing the number does not yield a sufficiently large improvement in clustering separation. Once the clustering is complete, the cluster most correlated with desirable movement behaviors is identified, and the centroid is defined as the 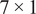 success-correlated coordination vector. In the case of the Kendama task, successful movements, those that result in the ball being caught in the cup, are defined as desirable behaviors.

**Figure 2.**
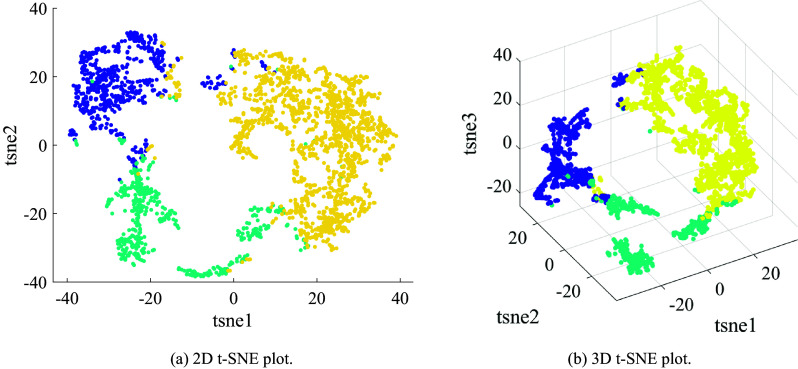
(a) and (b) show the t-SNE plots of the k-means clustering in 2D and 3D space respectively. The clusters are formed through unsupervised k-means clustering on unlabeled data. Three clusters separate the data without overfitting, shown in yellow, blue, and green. The one corresponding to the most number of successes, the yellow cluster in this example, is identified as the success-correlated cluster.

An example of the clustering process is shown in Figure 2 for 13 novice participants performing the Kendama task without any exoskeleton intervention for 200 attempts. These participants form the baseline control group as discussed in the following sections. The first principal coordinate is identified for all attempts across participants, and unsupervised clustering is used to separate the data. The within-cluster-sum-of-square (WCSS) was plotted for 2 to 10 clusters and an elbow was identified at 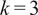 indicating that 3 clusters maximize the distance between the clusters while minimizing overfitting. Figure 2a and b show the t-SNE plots of the k-means clustering when 3 clusters are identified. The number of clusters corresponds to the maximum number of clusters before an increase in this number does not cause a significant decrease in clustering inertia. Next, the cluster correlated with the most number of successes is identified as cluster 3 shown in yellow. The centroid of this cluster is selected and the corresponding unit coordination vector is identified as the task-specific coordination behavior.

This preferred coordination can be calculated for any number of attempts both within and across participants. When used on attempts on the same task performed by different participants (as in Figure 2, the resulting coordination is a task-specific coordination behavior. When used on a single participant’s attempts after training, this coordination represents their learned coordination. Similarly, participant-specific initial coordination can be identified by clustering their initial attempts. While the first principal component may not describe all of the motion, for most comparisons presented in this paper, this coordination represents at least a majority of the motion ( 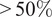 ). Next, we describe the exoskeleton control mode developed to constrain the human-robot combined system’s movement along or orthogonal to a desired coordination.

### 3.3. Exoskeleton control mode 1: fixed coordination constraint

An impedance-controlled force field is used to impose a desired coordination behavior at each joint. Details of the control mode can be found in Ghonasgi et al. (2023b), but we repeat the salient features here for completeness. The torque commands for the joint angle coordination control (JACC) are(2)

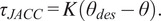



Given an arbitrary 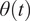 at time 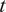 , we aim to identify the desired joint angles 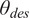 such that the desired joint coordination behavior, 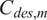 is maintained where 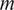 is can be any number between 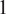 and 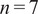 . Note that 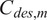 is constructed from the first 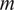 columns of the matrix 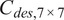 in Eq. 1 which are naturally ordered in decreasing order of contribution to movement variability. Using the full matrix 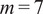 will result in no constraint as the participant will be constrained to their entire workspace. As each column is removed 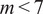 , the constrained space will lose a dimension, resulting in a stronger perception of the constraint and reduced workspace. The case considered in this paper is the most constrained version of this controller, where 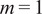 .

At time 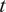 , we identify the 1-dimensional time-dependent signal relative to the desired coordination 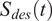 as(3)

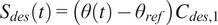



Given 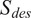 (the desired signal behavior), 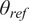 (the mean position of the movement for PCA), and 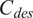 (the desired coordination matrix), the desired joint angles are(4)

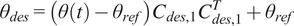



**Figure 3.**
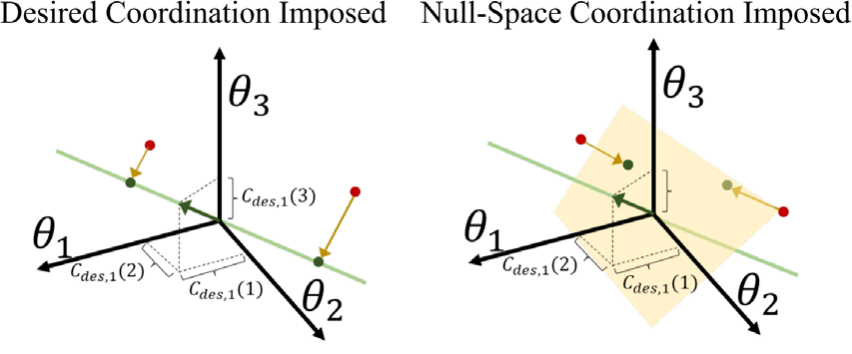
Visualization of imposing (left) the desired coordination (Ghonasgi et al., 2023b) and constraining to the null space of (right) the desired coordination for an example 3 degree of freedom system.

Note that if 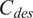 of size 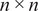 is used in Eq. 3, the corresponding 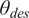 in Eq. 4 is the same as 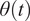 . By reducing the dimensionality of 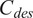 , we restrict the relative behaviors at each joint to a specific coordination. The calculated desired joint angles 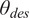 define the closest joint angle pose such that the desired coordination is satisfied. A simplified example of this coordination control is depicted in Figure 3 (left). For a human-robot system with three degrees of freedom, say the green line represents the desired coordination behavior. If the system moves away from a desired configuration (to one of the red points), the desired coordination assistance applies a force field to bring the system to the nearest desired configuration depicted by the yellow vectors.

**Figure 4.**
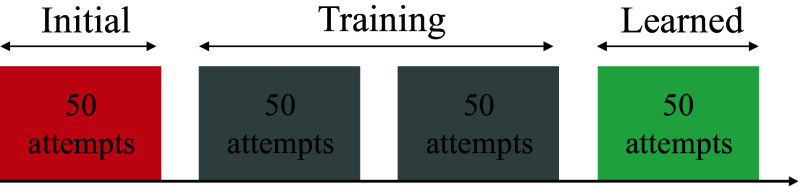
Experimental protocol followed for the current study. Each group of participants completed 200 attempts. The first 50 and last 50 were completed without kinematic constraints. Depending on the group, participants received different interventions during thw 100 training attempts.

It is important to note that a dynamic task like Kendama is likely to be complex and require more than one kinematic coordination or synergy to describe 90% of movement variance using PCA. However, to simplify control and enforce a single dominant coordination pattern, we used only the first principal component derived from participant attempts for the personalized coordination control, rather than attempting to reconstruct the full variance of the movement. Similarly, in our prior work, we identified expert behaviors that correlated with three principal components for the frontal plane (side-swing) strategy. However, only the first principal component is used for the current study as it describes the majority of the variability in the joint kinematics. While a simplified approach is explored for the purposes of this paper, future modifications could consider more complex multi-coordination approaches for novel task training interventions.

### 3.4. Exoskeleton control mode 2: null space coordination constraint

The null-space coordination intervention complements the desired coordination assistance intervention described in the previous section. Rather than constraining the robot’s movement to a desired coordination, the movement is constrained to the null space of the predefined coordination vector.

Thus, the desired joint angles may be calculated as(5)

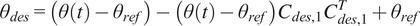

where 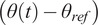 is the vector from the origin (centered on 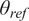 ) to the current configuration, and the second term is the yellow vector perpendicular to the coordination vector. The perpendicular component of 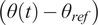 is then given by(6)

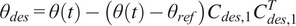



This coordination control is also depicted in Figure 3 (right). The null-space controller identifies the surface perpendicular to the desired coordination. For a 3-dimensional human-robot system, this null-space is a plane. The coordination null space assistance moves the system toward the nearest configuration on the null-space plane, represented by the yellow lines.

### 3.5. Experimental protocol

Our goal was to quantify the feasibility and effect of coordination-based interventions in dynamic task learning. We do this by quantifying the learning effects in terms of extrinsic and intrinsic performance metrics for the Kendama task while wearing the Harmony exoskeleton under different coordination-based interventions. We present results from a human subject study with a total of 43 participants (13 female, age 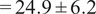 ) in four different training groups. In each group, participants trained on the frontal-plane strategy for the Kendama task for a total of 200 attempts while wearing the Harmony exoskeleton. The first 50 and last 50 attempts of the session were performed in the exoskeleton’s gravity support mode, where the robot compensates for its own weight, giving the sensation of moving in water due to the inherent inertia and friction. The first group (13 participants, 4 female), also referred to as the *Control* group, always used the gravity support mode throughout their training. Note that the data from this group of participants was collected prior to the exoskeleton-intervention groups and has been presented in prior work (Ghonasgi et al., 2022). The performance of this first group led to the construction of an expert-correlated frontal-plane kinematic coordination using the clustering method described earlier. We previously introduced a joint angle coordination control more (Ghonasgi et al., 2023b) which allows the Harmony exoskeleton to impose a set coordination behavior during movement. The current work focuses on leveraging these prior works (expert coordination and coordination-based constraint) as interventions for exoskeleton-based training. The stiffness of the impedance controller at each joint was set beforehand (as per Ghonasgi et al., 2023b) and was not varied across participants or tasks.

The second group (10 participants, 1 male left-handed, 2 female), referred to as the *preferred constraint* or PC group, trained with individual-specific coordination behaviors during the 100 training attempts. The individual-specific coordination is identified using the clustering method described earlier on the first 50 attempts for each participant, where they perform the task in gravity support mode. Each participant in this group is trained on a different individual-specific coordination behavior. The across-participant frontal-plane kinematic coordination identified from the control group is used to construct the intervention for the third and fourth groups. The third group (10 participants, 1 male left-handed, 4 female), referred to as the *fixed constraint* or FC group, trained with the task-specific coordination imposed during their 100 training attempts. For the second and third groups, the fixed coordination constraint control mode was used to impose the target coordination. The fourth group (10 participants, 1 male left-handed, 3 female), referred to as the *null-space constraint* or NC group, trained with the same task-specific coordination behavior but with the null-space coordination constraint control mode during training. All participants fell within 3 standard deviations for their arm measurements, except 1 in the null-space group, whose forearm was longer than expected but still under five standard deviations from the mean.

The experimental protocol was reviewed and approved by The University of Texas at Austin IRB (Study 1215). Participants were informed of the experiment protocol prior to the experiment and signed a consent form allowing the collection of experimental data. All data was collected in accordance with the approved protocol and the relevant COVID-19 guidelines on human-subject experimentation.

### 3.6. Outcome measures and analysis

Evaluation metrics for dynamic motor learning tasks can be categorized into extrinsic and intrinsic metrics (Magill & Anderson, 2010; Ghonasgi et al., 2021). Extrinsic metrics capture the outcome of the task performance, while intrinsic metrics capture the inherent properties of the execution (Ghonasgi et al., 2022). For the Kendama task, we select success rate, or number of successes in a block of 50 attempts, as the extrinsic performance metric. We define a second extrinsic metric called ‘close attempt rate’, equivalent to the number of attempts within a block of 50 that were close attempts. A close attempt is defined as one where the ball comes very close to the Kendama cup (within a radius of 5 cm, the sum of the radii of the bowl and ball with a small margin of error). All successes are close attempts, but not all close attempts are successes. The close-attempts rate is less sparse than the success rate metric. This metric allows us to compare performances across individuals with different learning rates in a relatively short training session. We hypothesize that the participants will learn the Kendama task in all conditions, but that there may be an effect of the training group type on the extent of performance improvement. As the fixed constraint group train with the task-specific coordination, we expect to see steeper improvement in this group compared to the others.

Three intrinsic metrics are also considered. First, the method described is used to identify the initial successful coordination for a given participant using only their initial block (first 50 attempts) and this coordination is compared to every attempt, referred to as the *distance to participant initial coordination.* Second, the same method as used to construct the first control mode is used to identify the learned successful coordination for each participant using only their learned block (final 50 attempts). This coordination is also compared to every other attempt at the task and the averaged results from a given block are referred to as the *distance to participant learned coordination.* Finally, the third metric compares joint angle coordination for each attempt to the task-specific coordination observed in the ‘Control’ group data, referred to as the *distance to task-specific coordination.* A lower distance indicates that the coordination for that attempt is similar to the coordination observed in the control participants learning the frontal-plane task.

We hypothesize that all participants will converge toward their participant-specific learned coordination as they train (H1). Parallelly, we expect that all participants will move away from their initial coordination, except those in the preferred constraint group, as these individuals will train with their initial coordination as the training constraint (relating to H2 and H3). Finally, we hypothesize that participants in the fixed constraint group will converge toward the task-specific coordination they train with, while the null-space constraint group will move away from this coordination (relating to H2).

### 3.7. Statistical analysis

Two-way repeated measures ANOVA is used to compare performance metrics averaged over the initial phase (first 50 attempts) and the initial training phase (attempts 51–100). This comparison is conducted to test whether the different training interventions affected the movement behaviors and corresponding outcomes. Next, two-way repeated measures ANOVA is used to evaluate the overall effect of training and cross effects of each intervention mode on participant-specific metrics. For comparison across intervention groups on intervention- and task-specific metrics, one-way ANOVA is used to compare learned performances across participants in all four intervention groups. The Shapiro–Wilk test is conducted to test for normality. When normality is violated, we will use a Wilcoxon rank-sum test to assess significance. Finally, when normality is not violated, one-tailed t-tests are conducted to reveal specific trends between different session phases and intervention groups. A statistically significant effect is identified by a p-value less than 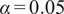 . The two factors are between-subject intervention type (control, fixed constraint, preferred constraint, and null-space constraint) and within-subject phase type (initial or learned).

## 4. Results

A total of 43 novices learned the virtual Kendama task under four different intervention conditions while wearing the Harmony exoskeleton. Of these, 13 participants are part of the control group, whereas the three intervention groups each consist of 10 participants. Note that experimental results from the control participants inform the design of interventions for two of the intervention groups, the fixed and null-space constraint groups. Participants in the intervention group are randomly assigned to one of the three groups described in the previous section.

### 4.1. Comparing initial performance to training

**Figure 5.**
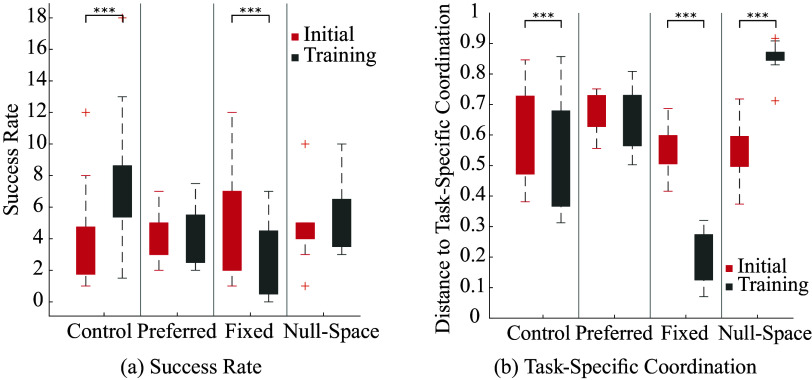
Comparing initial to training performance: (a) Success rate, and (b) distance to task-specific coordination. Significance is indicated as 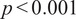 : ‘***’, 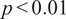 : ‘**’, 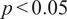 : ‘*’.

There is a statistically significant cross-effect of the intervention and phase on success rate ( 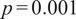 , effect size 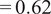 ), as seen in the left panel of Figure 5. One-tailed paired t-tests reveal that participants in the control group increased in success rate ( 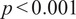 ) while those in the fixed constraint group decreased in performance ( 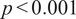 ). There is no significant difference in the null-space and preferred constraint groups. The same result is identified in the close attempts rate. As participants’ initial reference coordination is imposed during training for the preferred constraint group, we saw a statistically significant decrease in distance to their initial coordination during training ( 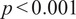 ). Further, participants in the null-space constraint group move away from their initially preferred coordination during the intervention ( 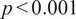 ). Analysis of the distance to task-specific coordination (depicted in the right panel of Figure 5) shows a significant effect of both intervention ( 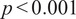 , effect size 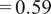 ) and training ( 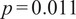 , effect size 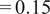 ), as well as a significant cross-effect ( 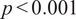 , effect size 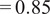 ). Specifically, we find that participants show a decrease in their distance to the task-specific coordination in both the fixed constraint ( 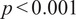 ) and the control ( 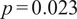 ) groups, whereas they show an increase in this distance in the null-space group ( 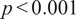 ). Note that as the fixed constraint imposes the fixed coordination, and the null-space constraint imposes the null space of the fixed constraint, these results validate the implementation of the controller.

### 4.2. Comparing initial to learned performance

This subsection compares the initial to the learned performance in participants across the four intervention groups.

#### 4.2.1 Extrinsic metrics

**Figure 6.**
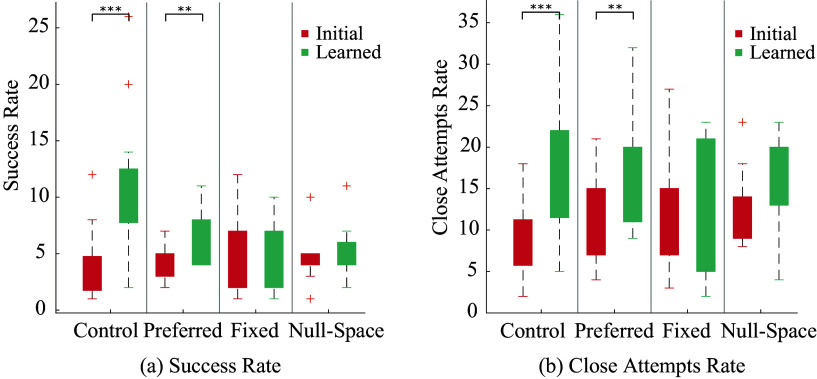
Extrinsic performance metrics comparison across the different intervention groups: (a) success rate and (b) close attempts rate.

Two extrinsic performance metrics are considered: the success rate (number of successes in a 50-attempt phase) and close attempt rate (number of attempts where the ball came close to the cup in a 50-attempt phase). The results are presented in Figure 6. The success rate results reveal a significant effect of the phase type across groups ( 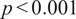 , effect size 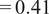 ) as well as a cross-effect of the intervention and phase ( 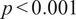 , effect size 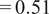 ). A comparison of close attempt rate reveals a similar effect of phase type ( 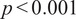 , 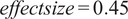 ) and cross-effect of phase and trial ( 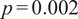 , 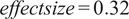 ). Post-hoc paired *t*-tests are conducted to compare initial and learned metrics to identify which specific intervention groups saw significant changes in performance. Using a one-tailed *t*-test, we tested for an increase in performance from the initial to the learned phase within each intervention group. The results show an improvement in success rate in the control group ( 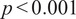 ) and in the preferred constraint group ( 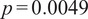 ). The fixed constraint group does not show statistically significant improvement in success rate after training. Data from the null-space constraint group failed the Shapiro–Wilk test of normality. Thus, an additional Wilcoxon rank-sum test is used. The result shows that participants in this group did not significantly improve their success rate either. It is worth noting that data from the fixed constraint group was also close to failing the normality test ( 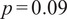 ). The success rate phase means are: control group = 7.9 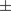 3.3, preferred constraint group = 2 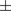 0.55, fixed group = 0 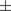 0.82, null-space group = 0.6 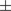 0.29. The close attempt rate improvement showed no statistical difference between the control, preferred constraint, and null-space constraints group, though the fixed constraint group improvement was significantly lower than the other three ( 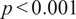 ).

#### 4.2.2 Intrinsic metrics

**Figure 7.**
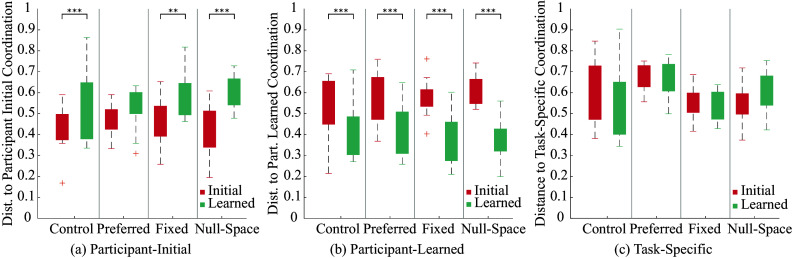
Intrinsic participant-coordination performance metrics within participants across intervention groups: (a) distance to initial coordination, (b) distance to learned coordination, (c) distance to task-specific coordination.

Three participant-specific intrinsic metrics of performance are compared from the initial to learned phase: distance to participant-specific initial reference, distance to participant-specific learned reference, and distance to the task-specific reference identified from the control group. The results are presented in Figure 7. In each case, the participant-specific or task-specific reference is identified using an unsupervised clustering method and using the centroid of the cluster as described in the methodology section. The ANOVA results show that there is a statistically significant effect of phase on the distance to both participant-specific learned reference ( 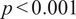 , effect size 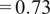 ) and initial reference ( 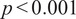 , effect size 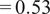 ) behaviors.

**Figure 8.**
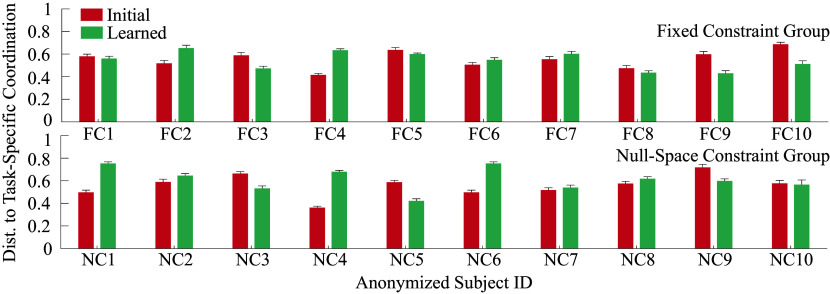
Participant-wise distance to task-specific coordination: Each pair of bars compares initial (red) versus learned (green) coordination distance for the 10 participants in the fixed constraint group (top) and null-space constraint group (bottom), respectively. Note that the subject IDs are anonymized and not necessarily in the order of data collection.

Additional t-tests reveal that participants tended to move away from their initial coordination behavior (increase distance) except in the preferred constraint intervention group ( 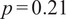 ). There was no statistically significant effect observed of the training phase or intervention on the distance to the task-specific coordination behavior. The participant-specific results are presented in Figure 8 for the fixed and null-space constraint groups. 6 out of 10 participants in the fixed constraint group show the expected decreasing trend in the distance to the task-specific coordination metric. Similarly, 6 out of 10 participants in the null-space group show a trend of moving away from task-specific coordination. The relatively small number of participants and the highlighted individualized trends indicate a need for longer interventions with a larger number of participants.

## 5. Discussion

Prior research (Bockemühl et al., 2010; Magill & Anderson, 2010; Ghonasgi et al., 2022) suggests that novel motor skill acquisition is accompanied by consistent coordination of joint kinematics. Our prior work demonstrated that such coordination behaviors are indeed learned over time as individuals learn a dynamic task. We also find evidence that experts converge on the same task-specific coordination behaviors as they learn. Further, assistance of a task and participant-specific coordination behaviors has been found to improve task performance for a straight-line reaching task (Ghonasgi et al., 2023b). These results motivate our interest in coordination-based exoskeleton control as a mode of assistance and training intervention for the human–robot system.

We selected the previously described virtual Kendama task as the testbed for our training environment. The dynamic nature of the task sets it apart from previously studied static reaching tasks (Proietti et al., 2017; Ghonasgi et al., 2023b). We also ensure that participants are relatively unfamiliar with the task to allow the observation of motor learning, further differentiating this study from previously conducted reaching studies. We explore the effect of training with three training interventions that constrain the participant either to their own observed behavior, to the coordination identified from the side-swing control group, or to the null space of the side-swing coordination. Specifically, we aimed to address three key questions relating to the feasibility and effect of the intervention: *1) Can participants perform and learn the dynamic task under a coordinaiton-based constraint during training?*, *2) Does learning with the constrain help or hamper task performance and learning?*, and *3) How does training with task-specific and participant-specific behavioral constraints affect the learned behavior?.*

The extrinsic metrics, success rate, and close attempts, both show statistically significant effects of the intervention on performance improvement from before to after training (Figure 6). Participants improve on the sparse success rate metric only in the preferred constraint and control groups, suggesting that introducing an unfamiliar coordination behavior affected the learning of the dynamic task. This finding partially supports H1, indicating that participants can learn the task under different intervention conditions. However, simultaneously, the lack of improvement in the remaining constraint groups suggests both interventions impeded learning (supporting H2). This effect is particularly pronounced in the fixed constraint group in the close attempts metric, but less in the null-space constraint group, suggesting further that the fixed constraint intervention was more detrimental to learning. These observations may be explained by the reduced exploration during training, thereby restricting participants from learning the dynamics of the environment well enough to improve overall performance outside the intervention. At the same time, the preferred constraint group receives a similar level of restriction to the fixed constraint group, but participants still improve their performance after training. This result suggests that the preferred coordination behavior identified in the initial phase of the session is familiar enough to participants that they do not suffer the same level of restriction as those in the fixed constraint and null space constraint groups.

It should be noted that the improvement in success rate in the control group is higher than that in the preferred constraint group, though the close attempt rates are equivalent. These results indicate that the coordination-based intervention during learning made the task harder for participants to learn. At the same time, the imposition of a weak (null-space) or personalized (preferred) constraint did not hinder learning as much as an unfamiliar strong constraint (fixed) (relating to H3). The three intervention modes may be ordered in terms of the corresponding impact on the improvement of extrinsic performance as preferred constraint 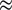 null-space constraint 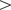 fixed constraint. This ordering may be inversely proportional to the effective task difficulty based on the observed extrinsic outcomes.

Three intrinsic performance metrics are considered as they pertain to the learned kinematic behavior we aim to affect. The first metric, distance to learned successful coordination behaviors has previously been established as an indicator of learning (Ghonasgi et al., 2022; Ghonasgi et al., 2023a). Specifically, a decrease in distance from before to after training suggests that participants learn to move in a more consistently coordinated manner. The same trend is observed in all intervention groups and the control group, indicating that although some participants may not have improved in terms of their extrinsic performance, they all converge to participant-specific coordination behaviors. Thus, all participants learned to move more consistently, if not more successfully, regardless of the constraints from the exoskeleton (relating to H1, Figure 7b). Second, we consider the effect of the training on the distance to a participant’s initial preferred kinematic coordination (Figure 7a). This preferred coordination is used to design the training intervention for the preferred constraint group, and we expected to see that participants would remain close to their initial coordination. The results show that participants in this group did not change their distance to initial coordination from the initial phase to the learned phase (relating to the effect of personalized intervention, H3). In contrast, participants in the other two intervention modes and the control group all move away from their initial coordination after training (relating to H2 and H3). Thus, participants tend to move away from their initial coordination as a result of training, but this change is affected by imposing the initial coordination during training. Third, we consider the distance to the task-specific learned coordination behavior identified by observing the control group (relating to H2 and H3). We expected the fixed constraint group participants to learn the task-specific coordination imposed during training. However, none of the three intervention groups shows statistically significant effects of training on this metric, comparing the initial to the learned phase attempts.

Taken together, these results allow us to answer the initially posed questions. The first question is answered readily: participants can learn a novel dynamic task under a coordination-based constraint, though the constraint may be detrimental to the magnitude of performance improvement. At the same time, we find that coordination-based interventions may prove to be a novel means of introducing task difficulty modulation for motor training. Even when participants do not improve in their extrinsic performance, they converge to a kinematic coordination behavior, suggesting they learn a non-optimal motor behavior. The answer to the second question can be split into two parts based on the results of the current study. First, participants learn to become more consistently coordinated in their joint behaviors, but there need not be a direct impact of the imposed coordination on the learned coordination behavior with this short training period. Second, the extent to which movement is constrained during training affects how difficult the task appears and how well learning translates from training to post-training. The imposition of familiar kinematic constraints did not make the task as difficult as constraining to an unknown coordination behavior. Finally, more than half the participants in the fixed constraint and null-space groups show the expected trends in the distance to task-specific coordination. The high inter-person variability suggests that the lack of significance may be due to short training periods, a small dataset of participants, and highly individualized learning trends across various types of learners. Other factors, such as experience with virtual reality, Kendama-like tasks, or high athletic skill could also result in variability in the effects of the exoskeleton’s intervention on the learned behaviors. Future exploration of such interventions that are aimed at teaching coordination behaviors should consider longer training sessions and multiple sessions to investigate the effect of the training, as well as controlling for other potential factors (such as familiarity with dynamic sports) in addition to ensuring task novelty.

These observations give us some key takeaways that should be considered when designing interventions for motor training in the future. First, while participants are able to perform dynamic tasks, the exoskeleton’s intervention, if not personalized, could result in an increase in task difficulty. Second, learning with a constraint typically resulted in decreased learning efficiency. Third, participant-specific training interventions may be more suitable for task learning than task-specific behaviors. At a high level, these observations may be explained as a consequence of the exploration-exploitation trade-off during training. Prior work indicates that pushing learners out of their comfort zone could enhance exploration, especially when directed towards desired behaviors (Komar et al., 2019; Hacques et al., 2021). Counterintuitively, our participants who were provided such constraints struggled the most to learn, suggesting that the selection of these constraints needs to be more nuanced. Specifically, participants in the control group are given the most freedom to explore during training, while those in the intervention groups are constrained. The interventions appear to significantly decrease exploration for the fixed and null-space groups so that participants do not learn the task dynamics as easily. However, interestingly, when the constraint is tailored to a participant’s own initial behavior, their exploration is not as significantly impacted. We know that participants tend towards certain participant-specific learned behaviors as they progress through the training (Ghonasgi et al., 2022; Ghonasgi et al., 2023a) and that when left to explore freely, they will move away from their own initial behaviors as seen in the results comparing the initial and learned attempt distances to the initial preferred coordination. Future iterations of this experiment could explore a null-space constraint using participant-preferred initial coordination behaviors as a mode of task training. Such an intervention would allow participants to more steeply converge on their own learned behaviors in the same training period.

Another limitation of the current study is the task that was selected, and the uncertainty of the generalization of our findings to other tasks. For example, the Kendama task requires two principal kinematic coordination behaviors to achieve success. The interventions discussed here may be challenging to extend to a task that requires more degrees of freedom for success or different coordination for different phases of the movement. Alternatively, the training intervention could be more effective in a dynamic task where a single coordination behavior is necessary for task success. Future work will use similar coordination-based interventions for training dynamic tasks with varying levels of complexity, as well as longer training sessions for tasks like Kendama. Further studies must also be conducted to identify how the time-independent coordination imposition correlates with the learning of a specific time-dependent behavior across participants trained with the same intervention.

An important application of such a training intervention is for the physical rehabilitation of impaired populations. Our prior work has shown that kinematic coordination can provide assistance in performing a reaching task. The coordination mode may be more suitable as an assistive intervention for participants who are unable to perform the task without any robot assistance. Participants suffering from neurological impairments, who thus cannot maintain a task-specific movement coordination, could potentially benefit from training with such an intervention.

## 6. Conclusion

This paper explores how a robotic exoskeleton could leverage joint-level coordination in the assistance and training of individuals with varying levels of ability. Our findings suggest that coordination-based intervention can have varying effects depending on the task and the individual, suggesting the need for personalization of intervention design. When applied to the broader context of exoskeleton-based motor training intervention design, this paper provides two key insights. First, interventions should be designed and modulated to ensure task difficulty is not increased artificially. Modulation of the intervention to support exploration early in the training, but encouraging exploitation in the latter phase, may be beneficial to the learning process. Second, personalization of the intervention is likely to result in larger benefits in the motor learning process compared to training with unfamiliar task-specific constraints. Together, these insights suggest that motor training interventions may benefit from personalization and adaptive design. Such adaptive motor training protocols, or curricula, could be key to exoskeleton-based motor training and physical rehabilitation in individuals post neurological injury, such as stroke.

Although certain robotic interaction modes can appear to be assistive for well-known tasks and movement behaviors, the same may not be true when training naive individuals to learn a dynamic task. In particular, our observations suggest that training only in a single training environment, thus restricting exploration of the available motor control space, can be detrimental to learning as it may not transfer well to the true task environment. However, participants may benefit from training under different intervention conditions that allow and even encourage certain types of exploration. This study brings to light the challenges of generalizing exoskeleton-based interaction for motor training and, thus, physical rehabilitation. Specifically, our results suggest that such training design should enable both exploration and exploitation of movement strategies for novice learners while allowing for personalization of the training interventions. Future work should thus explore the development of personalized curriculum-like motor training frameworks for enabling exoskeleton-based motor training.

## Data Availability

Data for this paper are available from the authors upon request and will be made accessible in compliance with the protocol approved by the IRB at the University of Texas at Austin.
